# PROTOCOL: Effectiveness of Educational Programmes to Prevent and Counter Online Violent Extremist Propaganda in English, French, Spanish, Portuguese, German and Scandinavian Language Studies: A Systematic Review

**DOI:** 10.1002/cl2.70042

**Published:** 2025-04-17

**Authors:** Felipe Pathé Duarte, João Pedro Ramos, Pedro Barbosa, Matteo Vergani, Cátia Moreira de Carvalho

**Affiliations:** ^1^ Research & Development Centre on Law and Society (CEDIS) NOVA School of Law – NOVA University of Lisbon Lisbon Portugal; ^2^ EPIUnit ITR Instituto de Saúde Pública da Universidade do Porto Porto Portugal; ^3^ Instituto de Ciências Biomédicas Abel Salazar Universidade do Porto Porto Portugal; ^4^ School of Humanities and Social Sciences – Deakin University Geelong Victoria Australia; ^5^ School of Law and Government Dublin City University Dublin Ireland; ^6^ Portuguese Institute of International Relations NOVA University of Lisbon Lisbon Portugal; ^7^ Institute of Security and Global Affairs Leiden University Leiden The Netherlands

## Abstract

This protocol outlines a systematic review that aims to understand the effectiveness of educational programmes, delivered both online and offline, designed to prevent and counter the effects of online violent extremist propaganda in multiple languages. The primary objective is to assess the impact of interventions on reducing violent extremist attitudes, beliefs and behaviours. A secondary objective is to identify key factors that influence the effectiveness of these interventions. Eligible studies will include randomised controlled trials and quasi‐experimental designs that evaluate interventions, such as media literacy initiatives, counter‐narratives, alternative narratives and gamified approaches. The analysis will synthesise outcomes using meta‐analysis and narrative synthesis, concentrating on attitudinal and behavioural extremism measures. By addressing a significant research gap, this review aims to provide actionable insights for developing educational strategies to mitigate online extremist propaganda's spread, impact and radicalising influence.

## 1 Background

### 1.1 The Problem, Condition or Issue

The use of online platforms – such as social media, social networks, content platforms, video games, gaming platforms and forums – for spreading violent extremist propaganda has been a growing trend, especially among young people (Alava 2017; Europol 2022a; Lakhani 2021; Rieger et al. 2020). Online extremist content, be it propaganda or peer‐to‐peer communications, can sway vulnerable individuals to adopt terrorists' ideologies and potentially participate in violent actions. Violent extremists take advantage of the diverse and innovative online ecosystem to produce and share online propaganda with the intention of radicalising and recruiting followers (Nicholson 2023; Holbrook and Taylor 2019). Online platforms also contribute to community building, in both online and offline realms (Davey 2021; Lakhani 2021; Nicholson 2023).

Governments and civil society have taken proactive measures to counter violent extremist online propaganda: examples are the ‘Jamal al‐Khatib’ by ‘Turn – Association for the Prevention of Violence and Extremism’, the DECOUNT online game, the ‘Redirect Method’ developed by Jigsaw, the Gen Next Foundation and others (Helmus and Klein 2018). These initiatives involve reducing the appeal of such content and raising awareness, amplifying community voices to challenge digital extremist narratives (Atamuradova and Zeiger 2021; European Commission 2015; Nicholson 2023).

This review assesses the effectiveness of both online and offline educational programmes in mitigating the radicalising effects of online extremist propaganda. It specifically focuses on violent extremist attitudes, beliefs, and behaviours. The aim is to identify the factors that contribute to the success of these interventions. Eligible studies must analyse the impact of educational programmes designed to reduce violent extremism, which includes physical or psychological violence driven by political, religious, ideological or social beliefs that pose a threat to public security. This includes efforts such as to diminish the appeal of extremist propaganda or to help individuals recognise what constitutes extremist content. The review adopts a comprehensive approach to capture the wide range of observed outcomes.

### 1.2 Key Concepts

#### 1.2.1 Propaganda

Propaganda, mainly through online platforms, shapes public perceptions and can drive individuals towards extremist ideologies. It is not merely about persuading others but about manipulating beliefs and emotions, often by presenting distorted facts or falsehoods (Frischlich et al. 2015; Hassan et al. 2018). Propaganda serves as a potent tool for radicalisation, simplifying complex issues into ideologically driven narratives that encourage action in support of violent extremism. The rise of digital media has amplified the reach and sophistication of extremist propaganda, enabling the targeted recruitment and radicalisation of vulnerable individuals across diverse ideological spectrums (Aly et al. 2017; Conway 2017; Winter 2015; Winter et al. 2020).

Propaganda is a form of communication intended to reshape the target audience's perceptions and provoke action in favour of the propagandist's ideological cause or viewpoint. Through storytelling, it strives to convince the public of the validity of the presented idea (Ellul 1990; Jowett and O'Donnel 2012; Hassan et al. 2018; Taylor 2003), while excluding other viewpoints. The propagandist tailors the narrative to influence opinion rather than objectively present facts (Jowett and O'Donnel 2012). Propaganda often utilises false or misleading information to manipulate beliefs and preferences for a specific purpose (Benkler et al. 2018).

Various forms of false or misleading information fall under the concept of ‘information disorder’ campaigns, which is the act of sharing or creating false information with or without the intent to cause harm (Rozgonyi 2018; Wardle and Derakshan 2017). Subcategories, such as misinformation, disinformation and malinformation, can sometimes overlap (Wardle 2024). Misinformation is false information that is disseminated without the intent to cause harm. Disinformation is deliberately created false information intended to harm individuals, social groups, organisations or countries. Malinformation is correct information that is factually based, but manipulated, and also used to inflict harm on individuals, social groups, organisations or countries – examples are hate speech, harassment and/or incitement to violence (Broda and Stromback 2024; Wardle and Derakshan 2017; UNDP 2018). Information disorder has the potential to polarise public opinion and propagate hate, providing an opportunity to extremists who support or use violence to achieve ideological, religiously inspired or political goals (Borum 2011; Fitzpatrick et at 2002; Pathé Duarte 2024; Wardle and Derakshan 2017).

#### 1.2.2 Propaganda and Radicalisation

Propaganda often promotes a simplistic worldview to reshape a target audience's cognitive and emotional perceptions of an issue and to encourage action in the interest of the propagandist (Jowett and O'Donnel 2012). Propaganda, in the form of recruitment messaging, follows a constant pattern: identifying the issue, proposing a solution and explaining why action is necessary and who should take it (Wilson 1973). The creation and dissemination of online propaganda might lead to radicalisation and violent extremism (Bjola and Pamment 2018; Holbrook and Taylor 2019; Maragkos and Maravelakis 2022; Nicholson 2023; Smith et al. 2020; Zeiger and Gyte 2021).

To understand how propaganda can radicalise, it is important to understand the process of radicalisation through which individuals become involved in or carry out acts of violent extremism. Radicalisation occurs when an individual increasingly adopts extremist beliefs and attitudes, and/or behaviours that support violence to achieve ideological, religious or political goals (Neumann 2013; Schmid 2013; Stephens et al. 2019). It involves developing attitudes that support the use of violence for a cause, and for a small minority (< 1%) of radicalised individuals, the execution of such violence (Wolfowicz et al. 2021).

This review will examine both cognitive and behavioural aspects of radicalisation (Calvert 2024; McCauley and Moskalenko 2017; Wolfowicz et al. 2019). McCauley and Moskalenko (2017) distinguish these two forms of radicalisation by separating them into two dimensions. The model is structured into two pyramids, one representing cognitive radicalisation and the other representing behavioural radicalisation, with multiple levels within each pyramid. The first, ‘Opinion Pyramid’, progresses from apathy at the base to full ideological commitment at the apex, including sympathisers and justifiers of extremist beliefs at intermediate levels. The second, ‘Action Pyramid’, illustrates escalating involvement in extremist behaviours, starting with passive support, advancing to low‐risk activism like propaganda dissemination, and culminating in high‐risk, violent actions at the top. Cognitive radicalisation entails the process of adopting an extremist ideology. Behavioural radicalisation entails joining a radical group online or engaging in offline violence. Individuals' thoughts or beliefs (cognitive radicalisation) may not always align with their actions (behavioural radicalisation), as radical attitudes and beliefs alone are not always indicative of violent behaviour.

In contrast to views that emphasise personal traits or psychological factors, some scholars, particularly from the field of Critical Security Studies or Social Movements Theory, highlight the significance of social networks and group dynamics in fostering radical beliefs. They argue that radicalisation cannot be fully understood through individual factors alone; rather, it is profoundly influenced by political, social and cultural contexts. These contexts shape how individuals encounter and internalise extremist ideologies (Bartlett and Miller 2012; Bigo 2008; Della Porta 2013; Jackson et al. 2011; Tilly 2004). Within this framework, radicalisation is often seen not just as a shift towards violent extremism but as a broader transformation of identity and beliefs, influenced by structural inequalities and social grievances.

#### 1.2.3 Online Violent Extremist Propaganda

Ideology plays a role in radicalisation: it can lure individuals into extremism through manipulation and propaganda, while also providing a sense of empowerment and purpose by aligning with a higher cause (Borum 2011; Kruglanski et al. 2014; McCauley 2009). Extremist ideologies tend to rely on a dichotomous ‘us vs. them’ perspective, which positions the ingroup as victims and the outgroup as aggressors (Farinelli 2021). This framework encompasses a variety of manifestations, including religiously motivated extremism as well as far‐left, far‐right, and other ideologies.

To effectively spread extremist propaganda, three essential factors must be in place: a compelling ideology, the receptive state of the propaganda recipient and a trustworthy messenger to effectively deliver the message to the audience (Ritzmann 2017). This content can be shared, reaching even those not actively seeking it (Frissen 2021; Roberts‐Ingleson and McCann 2023). Online platforms provide opportunities for seeking out information about extremist ideologies and creating connections with violent extremist groups. Algorithms might direct individuals towards increasingly extreme content (Baele et al. 2023; Ribeiro et al. 2020; Whittaker 2022). This can result in a proliferation of information silos and, consequently, more pronounced homogeneous social echo chambers (Iqbal et al. 2024; Roberts‐Ingleson and McCann 2023).

The consistent and immersive interaction with such content can significantly influence individuals towards radicalisation (Borum 2011). This process is complex and involves exposure and the interaction of personal vulnerabilities, social influences and the broader context. Motivated in part by psychological mechanisms, including powerful emotions, such as insecurity, shame and anger, these interactions can transform into feelings of resentment and hatred towards another group (Borum 2011; Conway 2017; Hassan et al. 2018; Higgins and Thomas 2019). Extremist propaganda often provides emotional and social benefits, such as a sense of belonging to a new family or brotherhood/sisterhood (Ritzmann 2017). To maximise its success, propaganda attempts to resonate with the pre‐existing truth perceived by the target audience. However, the facts are twisted to conform to the ideology of extremists (Erbschloe 2021; Schmid 2013).

Online violent extremist propaganda refers to materials aimed at radicalising, recruiting and inciting violence. These materials—such as videos, speeches and written articles—are carefully distributed across various online platforms to enhance their reach and effectiveness (Higgins and Thomas 2019; Nicholson 2023). Through staging, dissemination and consumption, violent online extremist propaganda aims to reach individuals online, potentially creating conditions for more radicalisation and recruitment (Conway 2017; Singer and Brooking 2018; Ullah 2017). It aims to target people who exhibit signs of cognitive openness, are experiencing emotional turmoil, seek a fresh start or are simply inquisitive (Al Attar 2019; De Marinis 2019; Whittaker 2022). Violent online extremist propaganda and dissemination involve communicating messages to an audience and making direct online contact once an individual shows interest in a particular online platform (Radicalisation Awareness Network [RAN] 2016).

As a result, extremist actors might be radicalised, recruited and mobilised online (Alava 2017; Jensen et al. 2018), posing a challenge to governments, security forces and civil society organisations (Conway 2017; Hiltunen et al. 2017). Educational programmes play a dual role in both preventing and countering online violent extremism. Educational initiatives are designed to build resilience, critical thinking and media literacy in at‐risk communities (UNESCO 2017). Prevention empowers individuals to engage critically with online content, helping to lessen the appeal of extremist ideologies. These programmes actively encourage values of tolerance, social cohesion and democratic participation, fostering a more resilient society (Macnair 2019). Countering online violent extremism targets individuals already exposed to extremist ideologies and seeks to challenge or disrupt their engagement with violent narratives. It often involves creating counter‐narratives that present alternative viewpoints to undermine extremist content (Helmus and Klein 2018; Nicholson 2023; Rosand and Winterbotham 2019).

### 1.3 The Intervention

In this review, we will focus on educational programmes aimed at countering and preventing online extremist propaganda of any type of extremist ideology. Many researchers and policy makers believe that education is an important component of the efforts of preventing and countering extremist propaganda, as well as challenging the risk factors that might draw individuals to extremist organisations (e.g., Sjøen 2023). Educational interventions comprise strategies that empower participants with knowledge and skills to navigate the online world without being drawn to extremist propaganda. These include but are not limited to:

– promoting media literacy among participants,
– employing persuasion and inoculation techniques against extremist propaganda,
– the dissemination of educational campaigns that involve the use of narrative, namely counter‐narratives and alternative narratives, to prevent and counter extremist propaganda.

These interventions have been implemented in diverse populations (e.g., children, adolescents, adults), forms (targeted and universal) and settings (online and offline). Some of the included interventions might be preventative, directed to people who are not involved in extremism, while others might aim to counter violent extremism for those who are already involved or attracted to extremist propaganda.

For example, an online game called Radicalise ‘was developed to entertain as well as to educate, and to test the principles of active inoculation in an experiential learning context’ (Saleh et al. 2024, 552) to help individuals identify the recruitment strategies through extremist propaganda. The rationale behind this game is the inoculation theory, which posits that, like a vaccine uses a weakened virus to build immunity, cognitive inoculation introduces a weakened argument with a refutation, building resistance to future persuasion (McGuire and Papageorgis 1962). Through this process, resistance is built to future persuasion by exposing individuals to weakened arguments and counter‐arguments, making their attitudes more resilient to later influence (McGuire and Papageorgis 1962). The *Radicalise* game is a 15‐min simulation of social media, exposing players to content resembling early extremist recruitment tactics (Saleh et al. 2024). In the game, players act as recruiters for a fictional extremist group, aiming to recruit a new member through social media to advance the mission of the group. To test this intervention, the authors implemented a 2 × 2 mixed randomised controlled trial (*n* = 291), including 135 participants in the treatment group who played Radicalise, and 156 participants in the control group who played Tetris, an unrelated game. The authors implemented the game in the United Kingdom and participants were recruited via a website (The full details, including data set and analysis of this intervention, can be found here: https://osf.io/48cn5/). This intervention demonstrated successful results, as participants enhanced their ability to evaluate potentially harmful social media content, improved their capacity to recognise factors that make individuals susceptible to extremist recruitment and reported increased confidence in identifying manipulation strategies (Saleh et al. 2024).

### 1.4 How the Intervention Might Work

With the rise of extremist propaganda being spread online, educational programmes dedicated to preventing and countering this phenomenon have increased in recent years (see e.g., Phillips et al. 2020; Davies 2018).

Critical media literacy is one of the intervention strategies designed to equip media users with the skills needed to effectively navigate the online environment. To achieve this, these interventions aim to promote awareness, reflection and empowerment (Schmitt et al. 2019). Awareness involves recognising the presence of extremist content and the possibility of encountering propaganda online. It includes understanding manipulation techniques—such as rhetorical and visual tactics—and how media operates (Schmitt et al. 2019). This awareness often leads to reflection, a metacognitive process that enhances self‐understanding and situational insight, supporting informed decision‐making (Sandars 2009). In critical media literacy, reflection means evaluating online content to identify potential extremist or propaganda elements. Empowerment then builds on awareness and reflection, fostering confidence in identifying manipulative messages, participating in social discussions and actively challenging extremism and prejudice. Empowered individuals can critically question content and express their own perspectives, ultimately enhancing overall awareness (Schmitt et al. 2019).

As awareness of propaganda increases, so does the ability to critically analyse it. This critical reflection on extremist content necessitates understanding its presence on the internet. Reflecting on extremist material enables individuals to actively engage with and counter such content, while also enhancing their awareness of others who have already taken a stand against online propaganda (Schmitt et al. 2019).

The Institute for Strategic Dialogue implemented a global educational programme called Extreme Dialogue, which creates films and campaigns featuring former extremists from various types of extremism (Phillips et al. 2020). The rationale behind this intervention is that participants engage with former extremists through video testimonies and group discussions, gaining exposure to perspectives they might not usually encounter. This safe space fosters reflection on the consequences of ideologically driven violence. Sessions start with impactful films, featuring former perpetrators and survivors, which are followed by interactive activities to deepen understanding (Institute for Strategic Dialogue 2022).

One of the most widely used online interventions involves the use of counter‐narratives or alternative narratives. For example, the ‘Jamal al‐Khatib’ campaign, developed by the civil society organisation ‘Turn – Association for the Prevention of Violence and Extremism’, aims to provide alternative narratives to Islamist propaganda by employing techniques, such as online street work and narrative biography (European Commission 2015). Another notable type of intervention is the ‘Redirect Method’, developed by Jigsaw (a subsidiary of Google's parent company Alphabet Inc.), in collaboration with the Gen Next Foundation, Moonshot CVE and others (Helmus and Klein 2018).

Braddock and Horgan (2016) define counter‐narratives as ‘narratives that challenge the core themes of other narratives’ (p. 386) and as a tool designed to ‘persuade individuals at risk of radicalization’ (p. 387). In this way, counter‐narrative strategies aim to challenge dominant narratives by offering individuals alternative social perspectives that differ from those presented by the dominant voice (Carthy et al. 2020).

The DECOUNT initiative is an online game that combines counter and alternative narratives with gamification against violent extremism (Pisoiu and Lippe 2022). Throughout the game, the player interacts with various characters, some of whom are part of extremist groups. These characters use specific framing techniques, as identified in frame analysis, to try to convince the protagonist to join their cause. For instance, in the female identitarian storyline, the protagonist is exposed to a frame that encourages patriotic activism, portraying her as a victim of sexual violence. She is then confronted with xenophobic and conspiratorial narratives that contradict each other, forcing her to decide whether to abandon her original political beliefs. The game is designed as a social media simulation, where the protagonist's news feed starts with posts from friends but gradually becomes flooded with extremist content as she befriends more individuals from extremist circles (Pisoiu and Lippe 2022).

Importantly, however, some strategies used in educational interventions, namely counter‐narratives, might have a backfire effect, as some counter‐narratives do not seem to work and might even be counterproductive (Carthy et al. 2020). Two potential adverse effects are exposing people to extremist narratives and the blowback effect, if the message or messenger is unsuitable, which can amplify terrorist arguments instead of diminishing them, causing resistance of those who are already radicalised (Zeiger and Gyte 2021). Another example of backfire effects of the strategies that are used in educational interventions is when campaigns against extremist narratives give extremists a more public platform to present their arguments, potentially highlighting their views rather than weakening them (Zeiger and Gyte 2021).

### 1.5 Why It Is Important to Do This Review

To the best of our knowledge, a systematic review on interventions to prevent and counter online extremist propaganda does not exist, after conducting a thorough search. However, we found seven systematic reviews on adjacent topics that we believe are worth mentioning to demonstrate the need to perform this proposed review.

One systematic review aimed to analyse how the internet and social media may provide a space for hate speech, cyberhate and terrorist purposes (Castano‐Pulgarín et al. 2021). The conclusion of this review showed that cyberhate can be exacerbated through the use of the internet, leading to social harm.

Two systematic reviews exist on the adjacent topic of how exposure to media and extremist online content might influence radicalisation. One sought to analyse the effects of media content risk factors on radicalisation (e.g., role of television coverage of terrorist attacks and news‐media bias to increase feelings of hostility) (Wolfowicz et al. 2022). The results of this review show that online exposure to radical content has a stronger correlation with radicalisation than other media content risk factors and had the most significant impact on behavioural outcomes. The other systematic review analysed how the internet and social media may be a space favourable to violent extremism (Hassan et al. 2018). This review showed that exposure to radical violent online content can be linked to extremist attitudes and increased risk of political violence among White supremacist, neo‐Nazi and radical Islamist groups, and that the Internet can play a crucial role in shaping decisions, which, combined with offline factors, influences decision‐making.

Additionally, there are three systematic reviews on the topic of detecting online extremism. The first aimed to provide a synthesis on how social media are useful for detecting radical groups (Adek et al. 2021). In this review, the authors sought to comprehensively analyse radical group detection strategies on social media platforms to understand the circumstances of online radicalisation, trends and gaps. The results indicate that over the past 10 years, researchers have extensively investigated the use of social media analytics to predict and identify online radicalisation. They have applied a combination of machine learning techniques and other tools, focusing on data sources, features, geolocation and language to detect cyber‐extremist activities. Gaikwad et al. (2021) conducted a review of online extremism detection techniques, concluding that there is a lack of proper datasets for the detection and classification of extremist propaganda disseminated online. The third systematic review aimed to provide a detailed analysis of research regarding online extremism in textual content (Aldera et al. 2021). This review analysed the definition of extremism in different contexts, and uncovered challenges and gaps in previous studies, revealing opportunities to enhance and build upon earlier findings. Specifically, the challenges identified point to the absence of a universally accepted definition of extremism, lack of commonly adopted data set and challenges associated with methodology (e.g., limitations of Artificial Intelligence and deep learning systems for detecting online extremism). One final systematic review assessed the effects of counter‐narrative interventions in individuals exposed to extremist narratives, which, if not countered, may promote a violent extremist action or belief system (Carthy et al. 2020). This review was focused on providing a synthesis of the effectiveness of counter‐narratives in the reduction of the risk of violent radicalisation. The distinction between this review and ours is that we will assess the effectiveness of educational interventions that employ a variety of strategies, including counter‐narratives, games and digital and citizenship literacy. In this sense, our review will produce an understanding of what types of educational interventions (including counter‐narratives) are more effective to prevent and counter online violent extremist propaganda and therefore might include some of the studies examined by Carthy et al. (2020).

Although the reviews presented above may be useful in providing evidence on the relationship between propaganda and extremist violence, and on the tools that may be effectively used to detect such propaganda, there are some gaps that should be addressed. The first gap is understanding how education can prevent and counter online violent extremist propaganda. In fact, some education systems have struggled to keep up with the digital age and how people can build resilience against online extremist propaganda (Phillips et al. 2020). Also, the power of education cannot be neglected, as education can have a relevant role in preventing and countering violent extremism, namely among youngsters who use the internet and might be exposed to online extremist propaganda (Institute for Strategic Dialogue n.d.). As such, educational programmes are essential in diminishing the influence and attractiveness of extremist propaganda by employing a wide range of strategies to equip citizens with skills to resist extremist propaganda. Besides, another strength of our systematic review is that it will take a step further and include a breadth of languages (English, French, Spanish, Portuguese, German, and Scandinavian languages) and the ability to compare different subtypes of educational programmes to prevent and counter online extremist propaganda, without restrictions in terms of population. After consulting with stakeholders who work in the field with real‐world needs (e.g., policymakers and practitioners), these were the languages in studies that would be most useful to understand the phenomenon under review.

## 2 Objectives

The primary objective of this review is to answer the following questions:

- How effective are the educational programmes employed to prevent and counter the radicalising impact of online extremist propaganda?
  This review will seek to understand the effectiveness of online and/or offline educational interventions to prevent and counter online violent extremist propaganda in English, French, Spanish, Portuguese, German and Scandinavian language studies. Violent extremist attitudes, beliefs and behaviour will be used as outcomes for effectiveness evaluation.
- What factors may influence the effectiveness of educational programmes to prevent and counter online violent extremist propaganda?

## 3 Methods

### 3.1 Criteria for Considering Studies for This Review

We will include studies on educational programmes to prevent and/or counter online violent extremist propaganda from any year and geographical focus. Eligible studies include those published in academic literature (e.g., peer‐reviewed journals) as well as grey literature (e.g., working papers and reports). Only studies that discuss and analyse primary data, whether published or unpublished, will be considered for inclusion. While review articles are not eligible, their references will be screened to identify primary sources that are potentially eligible for inclusion. Any post‐hoc alteration to the eligibility criteria will be clearly defined in the final report of the review.

#### 3.1.1 Types of Studies

Studies considered for inclusion in this review must provide data about a target group where participants are exposed to educational programmes to prevent or counter online violent extremist propaganda, and a comparison group. Prospective study protocols will be considered for inclusion if they match the inclusion criteria and are listed as ongoing studies in the appropriate reference lists.

The following study designs were selected because their levels of control over potential biases and confounding variables provide robust evidence, which is essential for evaluating the effectiveness of the interventions. Thus, we will include:

a. Randomised controlled trials with a pre‐defined method of randomisation for group allocation, whether experimental or control group (e.g., simple randomisation, block randomisation, etc.), that ensure comparability between groups. We will include business as usual, waitlist control groups, no intervention and also comparisons between alternative interventions. To ensure validity and reliability, studies should provide baseline post‐intervention measures or a brief follow‐up. No constraints will be imposed based on blinding decisions, but potential bias will be evaluated throughout the analysis.
b. Quasi‐experimental designs provided that face validity or covariate adjustment is included in the analysis and that the intervention and control groups are matched using approved techniques (such as propensity score matching, coarsened exact matching, exact matching). Unmatched control group designs with pre‐post intervention measures of the outcome, where the control group has face validity, will be included.

#### 3.1.2 Types of Participants

This review will be centred on languages, rather than countries, such that we will include primary studies written in the identified languages (i.e., English, French, Spanish, Portuguese, German and Scandinavian) regardless of the country where the interventions are implemented. Our aim is to summarise the findings of the interventions on the general public and subgroups of interest, such as people exposed to online extremist propaganda and/or at risk of being radicalised. In fact, anyone can be vulnerable to radicalisation; however, some might be more exposed or influenced by certain risk factors than others. According to Vergani et al. (2018), push factors such as relative deprivation and social exclusion; personal factors, like mental health issues and being young; and pull factors like charismatic leaders and extremist propaganda can contribute to putting someone more at risk of becoming radicalised. This is particularly concerning when young people are involved, as young people seem to be more vulnerable to extremist online propaganda (Pohl and Riesmeyer 2023). As such, educational interventions, with strategies like improving digital literacy and citizenship, raising awareness of extremist propaganda, or developing counter and alternative narratives, might reduce the impact of some risk factors and, hence, prevent violent extremism. This can be achieved through empowering people, both online and offline, with the necessary knowledge, skills and tools to challenge and deconstruct the messages of violent extremist groups (Zeiger and Gyte 2021).

Studies will not be excluded based on the participant's age, gender, marital status, education or socioeconomic background.

#### 3.1.3 Types of Interventions

We will include any educational intervention designed to prevent and/or counter online extremist propaganda of any kind of ideology, namely, religiously motivated extremism, far‐left and far‐right extremism, gender‐based extremism, ethno‐nationalist and separatist extremism. As such, eligible studies must review the effectiveness of an educational intervention intended to prevent and/or counter extremist propaganda.

Education is essential for shaping individuals and societies, providing the knowledge, skills and values needed for personal and societal progress (Chazan 2022). It encompasses a wide range of activities and processes that facilitate learning and nurture intellectual, social, emotional and physical growth, while transmitting knowledge and skills across generations, promoting intellectual, social, emotional and physical development (Chazan 2022). Beyond formal schooling, education includes informal and non‐formal learning, fostering critical thinking, creativity, problem‐solving and ethical decision‐making (Johnson and Majewska 2022). Informal learning is ‘the lifelong process by which every person acquires and accumulates knowledge, skills, attitudes and insights from daily experiences and exposure to the environment … Generally, [it] is unorganised and often unsystematic; yet it accounts for the great bulk of any person's total lifetime learning‐including that of even a highly “schooled” person’ (Coombs and Ahmed 1974, 3). On the other hand, non‐formal learning can be defined as ‘any organised, systematic, educational activity carried on outside the framework of the formal system to provide selected types of learning to particular subgroups in the population, adults as well as children’ (Coombs and Ahmed 1974, 8).

In recent years, the use of this type of interventions in preventing and countering violent extremism has become a significant focus of practice and research, as education is seen as a key component of a comprehensive approach to preventing and countering violent extremism, especially in strengthening cognitive defences against extremist propaganda (Aly et al. 2014; Novelli 2017).

Recently, there has been a unification of the concepts of Preventing Violent Extremism (PVE) and Countering Violent Extremism (CVE) under the combined term Preventing and Countering Violent Extremism (P/CVE). This integration appears logical; however, as the terms are frequently used interchangeably, it is challenging to differentiate between them in practice (Stephens et al. 2019). Thus, interventions designed to prevent violent extremism can be defined as preventative approaches that enable programmes to take a more comprehensive view of the underlying factors that contribute to vulnerabilities to violent extremism (UNDP 2018). These measures are put into practice with individuals who are not radicalised yet. As for interventions to counter violent extremism, they aim at mitigating the extremist threat through non‐coercive methods that directly address its root causes and focus mainly on countering the activities of individuals who are already radicalised and extremists (Sinai et al. 2019; UNDP 2018).

In this vein, this review will include studies that assess the effectiveness of educational programmes designed to prevent and/or counter online extremist propaganda and any outcome that meets the inclusion criteria. This review will include interventions implemented by civil society organisations, private sector companies, think tanks, academia and governments.

#### 3.1.4 Types of Outcome Measures

Eligible studies must report the educational programmes' effects on attitudinal or behavioural measures of violent extremism. The review will consider any attitude, belief or behaviour that indicates the use of violence (physical and/or psychological) motivated by a specific political, religious, ideological or social belief system, that presents as a threat to public safety. Violent extremist attitudes, beliefs and behaviour outcomes might be measured through self‐report instruments – for example, Extremism Scale, Pro‐Violence and Illegal Acts in Relation to Extremism Scale (PIARES, Ozer and Bertelsen 2018) – or qualitative methods, such as interviews, and observations of the participants.

Proxies of these constructs, such as sharing extremist propaganda or similar outcomes, will be considered as eligible. Our preliminary work indicates that a wide range of outcomes are measured in eligible studies, and thus we are defining these outcomes broadly and inclusively.

#### 3.1.5 Duration of Follow‐Up

Studies will not be excluded according to the length of the post‐intervention follow‐up. Outcomes measures at post‐intervention and any follow‐up time points will be included, and the studies will be categorised according to their follow‐up length (e.g., 3 months post‐baseline; 6 months post‐baseline; 1 year post‐baseline).

#### 3.1.6 Types of Settings

No restrictions will be placed on the type of setting in which the eligible intervention takes place.

### 3.2 Search Methods for Identification of Studies

#### 3.2.1 Electronic Searches

Relevant search terms on the topic were identified through a preliminary search and analysis of relevant studies. The search strategy was then created combining groups of search terms that reflect the (1) population, (2) intervention and (3) study design (see Table 1). Searches will be translated for each database and run across the Title, Abstract, Author Supplied Keywords and Indexing/Subject Term search fields. Below is the generic search strategy that will be used across databases and Appendix S1 includes the full syntax for Criminal Justice Abstracts. A comprehensive search strategy was defined to retrieve published and unpublished studies in multiple electronic databases, including:

– APA PsycArticles (ProQuest).
– APA PsycEXTRA (Ovid).
– APA PsycINFO (Ovid).
– Applied Social Sciences Index & Abstracts (ProQuest).
– Australian Criminology Database (Informit).
– Book Citation Index ‐ Social Sciences & Humanities (Web of Science).
– Conference Proceedings Citation Index ‐ Social Sciences & Humanities (Web of Science).
– Criminal Justice Abstracts (EBSCO).
– Cumulative Index to Nursing and Allied Health Literature (EBSCO).
– Dissertations & Theses Global (ProQuest).
– Epistemonikos (Epistemonikos).
– ERIC ‐ Educational Resources Information Center (ProQuest).
– International Political Science Abstracts (EBSCO).
– International Political Science Abstracts (ProQuest).
– SciELO Citation Index (Web of Science).
– Social Science Citation Index (Web of Science).
– Social Services Abstracts (ProQuest).
– Sociological Abstracts (ProQuest).

**Table 1:** Search terms.

Population	(bioterror* OR cyberterror* OR discriminat* OR extreme OR extremis* OR extreme‐left OR extreme‐right OR far‐right OR far‐left OR homophobi* OR ideolog* OR incel OR incels OR indoctrinat* OR islamis* OR islamophobi* OR jihadi* OR nationalis* OR neonazi* OR neo‐nazi* OR militant* OR militia* OR misogyn* OR propagand* OR racialist* OR racis* OR radical* OR salafi* OR separatis* OR transphobi* OR terror* OR violen* OR supremacist* OR supremacy OR xenophob*)
Intervention	(approach* OR campaign* OR counter OR counters OR counternarrative* OR ‘counter* narrative*’ OR discourag* OR educat* OR ‘fact check*’ OR ‘information integrity’ OR initiative* OR interven* OR inoculat* OR measure OR measures OR persuad* OR persuasion OR policy OR policies OR practice* OR prevent* OR proactive* OR program* OR project* OR reduc* OR scheme* OR service* OR stop OR stopping OR strateg* OR therap* OR train* OR treat*) N4 (disinform* OR ‘dis‐inform*’ OR extremis* OR ‘fake news’ OR ‘false inform*’ OR ‘false narrative*’ OR malinform* OR ‘mal‐inform*’ OR misinform* OR ‘mis‐inform*’ OR propagand* OR propagat* OR radical* OR terrori*)
Design	(allocat* OR ‘control group*’ OR ‘doubl*‐blind*’ OR evaluat* OR efficac* OR effective* OR experiment* OR pre‐post OR qualitative* OR quasi‐experiment* OR quasiexperiment* OR quasirandom* Or RCT OR random* OR ‘singl*‐blind*’ OR trial*)

#### 3.2.2 Searching Other Sources

A similar search will be conducted in grey literature repositories to include other literature not indexed in databases, to reduce publication bias and provide a balanced representation of existing evidence (Paez 2017). The search for eligible studies will include websites of institutions with reported research and/or communication in domains relevant to the current review, as per Table 2.

**Table 2:** Open access and grey literature sources.

Source	Website
Social Science Research Network	https://www.ssrn.com/index.cfm/en/
Open Science Framework	https://osf.io/registries
Center on Hate, Bias and Extremism (OntarioTech University)	https://socialscienceandhumanities.ontariotechu.ca/centre-on-hate-bias-and-extremism/index.php
Center for International Governance Innovation	https://www.cigionline.org/
Center for Strategic and International Studies	https://www.csis.org/
Commission for Countering Extremism	https://www.gov.uk/government/organisations/commission-for-countering-extremism
Council of Europe	https://www.coe.int/en/web/portal
Crime and Security Research Institute (Cardiff University)	https://www.cardiff.ac.uk/security-crime-intelligence-innovation-institute
Department of Homeland Security	https://www.dhs.gov/
European Foundation for South Asian Studies	https://www.efsas.org/
Institute for Strategic Dialogue	https://www.isdglobal.org/
International Centre for Counter‐Terrorism	https://www.icct.nl/
International Centre for the Study of Radicalisation	https://icsr.info
NATO	https://www.nato.int/
RAND Corporation	https://www.rand.org/
The Senator George J. Mitchell Institute for Global Peace, Security and Justice (Queen's University Belfast)	https://www.qub.ac.uk/Research/GRI/mitchell-institute/
United Nations Interregional Crime and Justice Research Institute	https://unicri.it/
Counter Extremism Project	https://www.counterextremism.com
The Soufan Center	https://thesoufancenter.org/intelbrief-2022-november-30/
National Consortium for the Study of Terrorism and Responses to Terrorism	https://www.start.umd.edu
Hedayah	https://hedayah.com
Moonshot	https://moonshotteam.com
Global Internet Forum to Counter Terrorism	https://gifct.org

Publications from selected journals published in the 6 months preceding the execution of this systematic search will be examined to identify potentially eligible studies that have not yet been indexed via the electronic search of academic databases. The following journals will be hand‐searched.

a. *Aggression and Violent Behavior*;
b. *Critical Studies on Terrorism*;
c. *Dynamics of Asymmetric Conflict*;
d. *Group Processes and Intergroup Relations*;
e. *Intelligence and Counter Terrorism*;
f. *International Journal of Conflict and Violence*;
g. *Journal for Deradicalization*;
h. *Journal of Interpersonal Violence*;
i. *Journal of Policing*;
j. *Journal of Politics*;
k. *Perspectives on Terrorism*;
l. *Police Quarterly*;
m. *Policing—An International Journal of Police Strategies and Management*;
n. *Policing and Society*;
o. *Political Communication*;
p. *Security Journal*;
q. *Sciences of Terrorism and Political Aggression*;
r. *Studies in Conflict & Terrorism*;
s. *Terrorism and Political Violence*.

Reviews identified during the search will be preserved for hand search of potentially relevant records from the reference list. Experts in the field will be contacted for pertinent information on the subject, especially the authors of studies that are included, in an effort to find unpublished research, studies that are being completed, and published studies that were missed by the database search (Saan et al. 2015). Moreover, a reference harvest will be conducted on the reference lists of the included studies.

### 3.3 Selection of Studies

#### 3.3.1 Pre‐Screening

The final search strategy will be inputted into the above‐mentioned selected electronic databases. Simultaneously, a comprehensive search of other relevant sources for eligible studies will be performed. The studies will be imported into EndNote and duplicates will be removed.

#### 3.3.2 Title and Abstract Screening

This review is closely interrelated with another being conducted by a similar team of authors (De Carvalho et al. in press, on misinformation, disinformation and malinformation [MDM]). One systematic search of electronic databases will be undertaken for both reviews, with the results of the search de‐duplicated first using EndNote, and a second round of de‐duplication will be completed in the information management software, EPPI‐Reviewer (Thomas et al. 2023).

The initial screening in EPPI‐Reviewer will be conducted using its machine learning algorithm for both reviews. A set of seed articles will be used to train the algorithm, allowing us to develop tailored classifiers. To begin, a subset of records will be manually classified into three categories (relevant to addressing MDM, relevant to educational interventions for reducing extremism/radicalisation and irrelevant) to assist the algorithm in learning what types of records are sought. This process enables the identification of records similar to those classified as eligible. The classifiers will subsequently sort records by their likelihood of inclusion, streamlining the screening process. By assigning a probability score to each record, the tool reduces manual effort and increases efficiency. Once trained, the classifiers can be applied to new records, automatically categorising them based on learned patterns. A recent study evaluating EPPI‐Reviewer found that the software significantly reduced screening workload by 9%–60% and offered good prioritisation accuracy (Tsou et al. 2020). We will screen documents using this software until EPPI‐Reviewer confirms that 95% of all potentially eligible records have been included. After this, we will review random samples of 30 additional records at a time until no more eligible records are found.

Studies retained at this stage will be assessed for their relevance to each of the respective reviews, that is, studies related to educational interventions will undergo full‐text assessment for inclusion in Duarte et al., while studies concerning MDM will proceed to full‐text screening in De Carvalho et al.

The screening of titles and abstracts of records using EPPI‐Reviewer will be the initial step in determining the eligibility of the study. This will allow for the removal of studies that are not relevant or eligible for inclusion (such as book reviews and non‐empirical research, as well as studies in different languages or not focusing on educational interventions to counter or prevent violent extremism). The AI function will be utilised solely for Title and Abstract screening.

#### 3.3.3 Full‐Text Screening

After the first screening phase, documents for all studies not deemed ineligible will be retrieved and added to EPPI‐Reviewer. The authors of studies that cannot be retrieved will be contacted to facilitate access to the studies. All documents will be independently reviewed by two members of the research team, with conflicts about eligibility being resolved through consensus with other members. In this sense, documents deemed ineligible by the two independent reviewers will be assigned a previously determined numerical code that corresponds to the rationale for exclusion, according to the codebook developed. The use of the codebook will help maintain consistency among reviewers, according to the following exclusion criteria: (1) study not available; (2) empirical study not on online extremist propaganda; (3) ineligible study design; (4) study language other than English, French, Spanish, Portuguese, German and Scandinavian; (5) study reports outcome measures outside the eligible ones.

#### 3.3.4 Data Extraction and Management

Data from each included study will be independently coded by two reviewers with the intent of summarising the information from each study, according to the following categories: (1) Author and year of publication; (2) Study design; (3) Study location; (4) Study setting; (5) Participants' baseline characteristics; (6) Sample size; (7) Strategies (such as media literacy or counter‐narratives); (8) Facilitators (such as teachers or psychologists); (9) Setting (such as universities or community); (10) Duration of the intervention; (11) Outcomes and (12) Effect size data. To enhance the quality of information retrieval, the extraction form has been piloted on a small, diverse sample of seed studies. Following extraction, data will be compared and harmonised before synthesis.

#### 3.3.5 Assessment of Risk of Bias

The risk of bias will be assessed according to Cochrane's RoB 2 for randomised trials (Sterne et al. 2016). Two members of the study team will independently evaluate the included studies based on each of the tool's subgroups and code them as ‘high’, ‘unclear’ or ‘low’. Any disagreement between researchers will be identified and resolved through consensus or via a third member (if a consensus cannot be achieved). Authors of ‘unclear’ studies will be contacted to provide further information in hopes of filling existing information gaps.

The revised Joanna Briggs Institute critical appraisal tool for the assessment of risk of bias for quasi‐experimental studies (Barker et al. 2024) will be used to ascertain the risk of bias in the design, conduct and reporting of these studies.

A summary table and figure, respectively, will be used to show the risk of bias between and within studies. There will be a brief examination of the possible ramifications in the discussion section of the results.

#### 3.3.6 Measures of Treatment Effect

We will undertake descriptive analyses on variables of interest from all included studies to give information on: (1) Study participant characteristics (e.g., subgroups of interest; age; gender; ethnicity; income level; language or idiom) and (2) educational programme characteristics (e.g., type of intervention; duration of intervention; setting).

Following descriptive analysis, we will calculate the effect sizes for each outcome of interest in each of the included studies. A meta‐analysis will be carried out only when two or more similar effect sizes are available, using a random effects model. Outcomes related to violent attitudes, beliefs or extremist behaviours may be presented as either continuous variables (e.g., number of detentions) or binary variables (e.g., participants who demonstrate violent extremist attitudes), depending on the measures employed and how the authors process the raw data.

The primary measure for the outcomes of this review will be logarithmic Odds Ratios (logOR), paired with 95% confidence intervals (95% CI). A logOR value significantly above 0 will indicate a positive impact of the educational intervention in preventing and countering online violent extremist propaganda, while a value significantly below 0 will reflect a negative impact. A logOR value of 0 will imply no effect from the intervention. When the outcome is reported as continuous, Cohen's *d* and 95% CI will be considered as the primary measure, thereafter converted into logOR, using Cox and Snell's method, by multiplying Cohen's *d* and its standard error by 1.65, assuming a normal distribution and equal variances across groups (Anzures‐Cabrera et al. 2011).

#### 3.3.7 Unit of Analysis Issues

Units of analysis may include individuals, groups and communities exposed to community‐based educational programmes designed to prevent and/or counter online extremist propaganda of any kind of ideology, namely, religiously motivated extremism, far‐left and far‐right extremism, gender‐based extremism, ethno‐nationalist and separatist extremism. In multi‐arm studies or studies with more than two groups, we will follow the approach proposed by Higgins and Thomas (2019), addressing the three key challenges associated with such designs. Specifically, we will determine which intervention groups are relevant to the systematic review, identify those suitable for a particular meta‐analysis and decide how to include multiple groups if more than two are deemed relevant. To address unit‐of‐analysis issues, particular attention will be given to the possibility of including multiple groups from a single study (Higgins and Thomas 2019, Chapter 23, 569). We will further consider using Robust Variance Estimation, taking into account the decision tree posited by Pustejovsky and Tipton (2021). R software will be used, through a combination of metafor, robumeta and clubSandwich packages.

Another potential issue with the unit of analysis relates to clustering. Given the scope, we do not expect clustering to be a significant concern, as the interventions included are generally implemented at the individual level. However, due to the diversity of settings − particularly in school or university environments − there remains a possibility of unit of analysis issues arising from clustering. To address this, we will apply the approach described by Higgins and Thomas (2019), through an estimate of the intracluster (or intraclass) correlation coefficient (ICC), combined with data gathered from the extraction form. If the included study does not directly report the ICC, an external estimate will be sourced from similar studies (Higgins and Thomas 2019, Chapter 23, 569).

#### 3.3.8 Criteria for Determination of Independent Findings

The present review may encounter primary studies reporting multiple effect sizes, such as multiple conceptually analogous outcomes, multiple measures of the same outcome, outcomes measured at different time points − for which we plan to include data from all time points across all documents in the analysis − or report results for different overlapping subgroups. This multiplicity introduces statistical dependency when the same participants contribute to multiple effect sizes, which, if ignored, can produce misleading meta‐analytic results. Addressing these dependencies appropriately is critical, as the chosen methods can substantially impact the findings of the meta‐analysis.

To address this issue, we plan to adopt the approach proposed by López‐López et al. (2018). This involves assessing the type and extent of multiplicity in the initial database of effect sizes, focusing on their combinability (López‐López et al. 2018). Combinability decisions include statistical considerations, such as evaluating whether different effect size metrics can be converted for analysis under appropriate assumptions (López‐López et al. 2018). Additionally, non‐statistical factors must be assessed, particularly the degree of similarity among studies, to determine whether they are sufficiently comparable for meaningful statistical combination (López‐López et al. 2018). After careful consideration of the characteristics of the effect sizes, we will apply the decision tree proposed by López‐López et al. (2018) to determine the appropriate procedures for managing each identified type of multiplicity.

#### 3.3.9 Assessment of Heterogeneity

The choice to undertake a meta‐analysis will be decided based on whether two or more studies are relevant to pooling (i.e., based on an assessment of whether participants, intervention, comparison and outcomes are sufficiently similar to ensure a meaningful result; Ryan 2020). Heterogeneity will be assessed based on: (1) Cochran's *Q* statistics; (2) the *I* ^2^ statistic and (3) *τ* ^2^. We will qualitatively discuss the methodological differences between studies and present a narrative synthesis of conclusions to highlight inconsistencies.

#### 3.3.10 Assessment of Reporting Biases

Publication bias will be assessed using funnel plots, which display study‐level mean effect sizes and 95% confidence intervals for the included studies to provide the opportunity for visual analysis of the precision of the estimated effect sizes, detection of studies with extreme effects and information regarding the heterogeneity of studies. The research team will analyse other reporting biases and describe them through narrative synthesis, in addition to those identified during critical appraisal (see Assessment of risk of bias).

#### 3.3.11 Data Synthesis

Where possible, that is, at least two methodologically comparable studies, we will synthesise the review results through a meta‐analysis in R using the dmetar package. A random effects model will be employed, allowing us to generate the pooled estimate while adjusting for both within‐ and between‐study variance. Multiple meta‐analyses will be conducted to assess the effectiveness of educational programmes aimed at preventing or countering online extremist propaganda, regardless of ideological basis. We will present the outcomes of interest along with a forest plot showing pooled effect sizes for each association, including 95% confidence intervals. For outcomes that cannot be combined in the meta‐analysis due to incompatible conceptual frameworks, we will report standardised single effect sizes.

For the secondary objective, we will conduct a narrative synthesis of the factors that influence the effectiveness of the educational programmes, as a means to identify and list the facilitators and barriers to implementation reported.

#### 3.3.12 Subgroup Analysis and Investigation of Heterogeneity

If the retrieval and review process results in a sufficient number of included studies, we will plan to perform several moderator analyses to determine potential effect size heterogeneity sources. Potential moderator categories may be related to research design (i.e., sampling approach – voluntary vs. non‐voluntary; follow up – no follow‐up vs. follow‐up); research setting (community vs. school interventions) and participant characteristics (i.e., gender – male vs. female; age ‐ older vs. younger individuals; area of residence – rural vs. urban; etc.). Additional post hoc moderator analyses of multiple moderators may be implemented. In our final report, these stages will be explicitly designated as post hoc analysis. We will run separate meta‐analytic models for each moderator with a minimum of two studies with visual representation through a forest plot.

If enough data is present to assess these effects, for categorical moderator analysis we will use the analogue‐to‐the‐ANOVA method (Lipsey and Wilson 2001), while for continuous moderator or analysis of multiple moderators will be conducted using meta‐regression (Higgins et al. 2020).

#### 3.3.13 Sensitivity Analysis

Sensitivity analysis will be performed to assess the robustness of the meta‐analysis and investigate the potential impact of outliers. The leave‐one‐out approach (i.e., removing one study from the data set at a time and reanalysing the results) will be used to evaluate the possible differences in the pooled estimate following the exclusion of specific studies. Studies with larger sample sizes, larger magnitudes of associations or with a higher risk of bias are expected to influence the pooled estimate. However, no studies will be omitted based on the results of the risk of bias assessment. Each study will be grouped according to risk of bias subcategories provided by the risk of bias assessment tool.

## Conflicts of Interest

The authors declare no conflicts of interest.

## Preliminary Timeframe

We will submit the first draft of the review with completed MECCIR Conduct and Reporting Checklists on 01 February 2025.

## Plans for Updating This Review

The authors commit to revising the review if there are enough new studies and sufficient funding to support this undertaking within a 5‐year timeframe.

## Supporting information


**Appendix 1. Example Search Strategy**.
Appendix 2. Structured Extraction Form.
