# Supplementary material for: PROTOCOL: Effectiveness of Educational Programmes to Prevent and Counter Online Violent Extremist Propaganda in English, French, Spanish, Portuguese, German and Scandinavian Language Studies: A Systematic Review
Source: Campbell Syst Rev. 2025 Apr 17;21(2):e70042. doi: 10.1002/cl2.70042 (PMC12004397; doi:10.1002/cl2.70042)
Supplement: Supplementary file 1 — Appendix 1. Example Search Strategy. Appendix 2. Structured Extraction Form. [file CL2-21-e70042-s001.docx]

**Supporting Information**

Appendices

**Appendix 1. Example Search Strategy**

Platform/Database: Criminal Justice Abstracts, via EBSCO (search date: 26 October 2024)

| **#** | **Searches** |
| --- | --- |
| S4 | S1 AND S2 AND S3 |
| S3 | TI (allocat* OR "control group*" OR "doubl*-blind*" OR evaluat* OR efficac* OR effective* OR experiment* OR pre-post OR qualitative* OR quasi-experiment* OR quasiexperiment* OR quasirandom* OR RCT OR random* OR "singl*-blind*" OR trial*) OR AB (allocat* OR "control group*" OR "doubl*-blind*" OR evaluat* OR efficac* OR effective* OR experiment* OR pre-post OR qualitative* OR quasi-experiment* OR quasiexperiment* OR quasirandom* OR RCT OR random* OR "singl*-blind*" OR trial*) OR KW (allocat* OR "control group*" OR "doubl*-blind*" OR evaluat* OR efficac* OR effective* OR experiment* OR pre-post OR qualitative* OR quasi-experiment* OR quasiexperiment* OR quasirandom* OR RCT OR random* OR "singl*-blind*" OR trial*) OR SU (allocat* OR "control group*" OR "doubl*-blind*" OR evaluat* OR efficac* OR effective* OR experiment* OR pre-post OR qualitative* OR quasi-experiment* OR quasiexperiment* OR quasirandom* OR RCT OR random* OR "singl*-blind*" OR trial*) |
| S2 | TX ((approach* OR campaign* OR counter OR counters OR counternarrative* OR "counter* narrative*" OR discourag* OR educat* OR "fact check*" OR "information integrity" OR initiative* OR interven* OR inoculat* OR measure OR measures OR persuad* OR persuasion OR policy OR policies OR practice* OR prevent* OR proactive* OR program* OR project* OR reduc* OR scheme* OR service* OR stop OR stopping OR strateg* OR therap* OR train* OR treat* ) N4 (disinform* OR "dis-inform*" OR extremis* OR "fake news" OR "false inform*" OR "false narrative*" OR malinform* OR "mal-inform*" OR misinform* OR "mis-inform*" OR propagand* OR propagat* OR radical* OR terrori*)) |
| S1 | TI (bioterror* OR cyberterror* OR discriminat* OR extreme OR extremis* OR extreme-left OR extreme-right OR far-right OR far-left OR homophobi* OR ideolog* OR incel OR incels OR indoctrinat* OR islamis* OR islamophobi* OR jihadi* OR nationalis* OR neonazi* OR neo-nazi* OR militant* OR militia* OR misogyn* OR propagand* OR racialist* OR racis* OR radical* OR salafi* OR separatis* OR transphobi* OR terror* OR violen* OR supremacist* OR supremacy OR xenophob*) OR AB (bioterror* OR cyberterror* OR discriminat* OR extreme OR extremis* OR extreme-left OR extreme-right OR far-right OR far-left OR homophobi* OR ideolog* OR incel OR incels OR indoctrinat* OR islamis* OR islamophobi* OR jihadi* OR nationalis* OR neonazi* OR neo-nazi* OR militant* OR militia* OR misogyn* OR propagand* OR racialist* OR racis* OR radical* OR salafi* OR separatis* OR transphobi* OR terror* OR violen* OR supremacist* OR supremacy OR xenophob*) OR KW (bioterror* OR cyberterror* OR discriminat* OR extreme OR extremis* OR extreme-left OR extreme-right OR far-right OR far-left OR homophobi* OR ideolog* OR incel OR incels OR indoctrinat* OR islamis* OR islamophobi* OR jihadi* OR nationalis* OR neonazi* OR neo-nazi* OR militant* OR militia* OR misogyn* OR propagand* OR racialist* OR racis* OR radical* OR salafi* OR separatis* OR transphobi* OR terror* OR violen* OR supremacist* OR supremacy OR xenophob*) OR SU (bioterror* OR cyberterror* OR discriminat* OR extreme OR extremis* OR extreme-left OR extreme-right OR far-right OR far-left OR homophobi* OR ideolog* OR incel OR incels OR indoctrinat* OR islamis* OR islamophobi* OR jihadi* OR nationalis* OR neonazi* OR neo-nazi* OR militant* OR militia* OR misogyn* OR racialist* OR racis* OR radical* OR salafi* OR separatis* OR transphobi* OR terror* OR violen* OR supremacist* OR supremacy OR transphobi* OR xenophob*) |

Appendix 2. Structured Extraction Form

Eligible studies that are object of full text review will be first organised alphabetically, thereafter coded with a unique alphanumerical code preceded by a # (e.g, #01, #02, …). This code will be the Report ID for the purposes of extraction. If the study does not report the required information, the corresponding field in the structured coding form should be completed with “Unclear/Absent.”

1. General characteristics

| a. Study ID  (surname of first author + year of publication) |  |
| --- | --- |
| b. Report ID |  |
| c. ID of Person Extracting Data |  |
| d. Date form completed  (dd/mm/yyyy) |  |
| e. Reference Identifier  (e.g., DOI, URL, …) |  |
| f. Publication Type  (e.g., peer-reviewed journal article, dissertation/thesis, conference proceeding, book chapter…) |  |
| g. Authors |  |
| h. Language | English  French  Spanish  Portuguese  German  Scandinavian  Other: __ |
| i. Country |  |
| j . Funding? | Yes  No  No information |
| j. 1. Funding details |  |
| Notes: | |

II.Study Details

| a. Study Characteristics | | Location in text or source  (page and paragraph/fig/table/etc.) |
| --- | --- | --- |
| a.1. Study Design | Experimental  Quasi-experimental |  |
| a.2. Factorial Design  (e.g., number of intervention groups x number of control groups) |  |  |
| Notes: | | |

| b. Participants | | | | Location in text or source  (page and paragraph/fig/table/etc.) | |
| --- | --- | --- | --- | --- | --- |
| b.1. Description (age, gender, region, language) |  | | |  | |
| b.2. Recruitment Method |  | | |  | |
|  |  | | |  | |
|  |  | | |  | |
| b.3 . Baseline imbalances |  | | |  | |
| b.4 . Withdrawals and exclusions |  | | |  | |
| b.5 . Sample Size Calculation | Yes  No | | |  | |
| b.6 . Sample Size  (add “comparison” columns depending on the number of arms in the study) | | | | | |
| Number of participants | | Intervention | Comparison | | Total |
| Recruited | |  |  | |  |
| Consented | |  |  | |  |
| Began the intervention | |  |  | |  |
| Completed the intervention | |  |  | |  |
| Completed follow-up  (if applicable) | |  |  | |  |
| b.7 . Sample Features  (add “comparison” columns depending on the number of arms in the study) | | | | | |
| Number of participants | | Intervention | Comparison | | Total |
| Gender (% male) | |  |  | |  |
| Age (M, SD) | |  |  | |  |
| Ethnicity | |  |  | |  |
| Religion | |  |  | |  |
| Notes: | | | | | |
|  |  |  |  |  |  |

| c. Intervention | | Location in text or source  (page and paragraph/fig/table/etc.) |
| --- | --- | --- |
| c.1. Allocation | Randomised  Matched  Score-Based  Other: _____________ |  |
| c.1.1. Additional information |  |  |
| c.2. Unit of allocation | Individual  Cluster  Other: ____________ |  |
| c.3. Setting | Community  University  /School  Online  Other: _________ |  |
| c.4. Designation  (include verbatim designation of the intervention group) |  |  |
| c.4.1. Theoretical Framework  (include key references) |  |  |
| c.4.2. Description of utilised strategies  (with sufficient detail for replication e.g., content, components, …) |  |  |
| c.4.3. Duration (in days, from recruitment to follow-up)  Obs. If intervention/comparison are conducted in a single moment please fill in with “0”. |  |  |
| c.4.4. Facilitator Characteristics  (include number, profession, training, and other relevant information) |  |  |
| c.4.5. Co-interventions | No  Yes. Which? ________________  _______________________  Not enough information |  |
| c.4.6. Delivery Mechanism  (means, intensity, fidelity) |  |  |
| c.4.7. Compensation for Participation | No  Yes. Which? ________________  _______________________  Not enough information |  |
| c.4.8. Cost of Intervention | No  Yes. Which? ________________  _______________________  Not enough information |  |
| Notes: | | |

(repeat table from c.4. onwards according to the number of interventions and comparison groups defined in a.2.)

| d. Outcome | | Location in text or source  (page and paragraph/fig/table/etc.) |
| --- | --- | --- |
| d.1. Outcome | Violent Extremist Behaviour  Violent Extremist Attitudes  Both  Proxies? |  |
| d.1.2. Additional information |  |  |
| d.2. Time-points measured  (select all that apply) | Pre  Post  Follow-up |  |
| d.2.1. Number of follow-ups |  |  |
| d.2.2. Follow-up time-points |  |  |
| d.3. Measurement | Self-report  Interview  Other: ____________________ |  |
| d.4. Measurement Tool |  |  |
| d.4.1. Validity |  |  |
| d.4.2. Reliability |  |  |
| d.4.3. Upper and lower limit |  |  |
| d.4.5. Thresholds |  |  |
| d.4.6. Interpretation |  |  |
| d.4.7. Missing data imputation | Listwise  Pairwise  Imputation by the mean/mode  Single regression  Dummy variable  No information  No missing data  Other: ______________________ |  |
| d.4.8. Power achieved |  |  |
| d.4.9. Statistical Significance | Yes  No |  |
| d.4.10. Direction of the outcome | Positive  Negative  No change |  |
| d.4.11. Group favoured  (refer to designations from c.4.) |  |  |
| d.4.11. Main conclusions |  |  |
| Notes: | | |

(when more than one outcome measured, repeat table from d.2. according to the number of outcomes.)

| e. Effect size | | Location in text or source  (page and paragraph/fig/table/etc.) |
| --- | --- | --- |
| e.1. Total sample for the outcome |  |  |
| d.1.1. Total sample for treatment group |  |  |
| d.1.2. Total sample for control/comparator |  |  |
| (add lines corresponding to the number of control/comparator groups [d.1.3., d.1.4., …) | | |
| d.2. Attrition | Yes  No |  |
| d.2.1. Attrition details |  |  |
| d.3. Raw effect size (value) |  |  |
| d.4. Standardized effect size | Yes  No |  |
| d.4.1. Value |  |  |
| d.5. Measure used | Mean  Standard Deviation  Standard Error  Proportion/Frequencies  Other: _________________ |  |
| d.5.1. Mean value (treatment) |  |  |
| d.5.2. Mean value (control/comparison) |  |  |
| (add lines corresponding to the number of control/comparator groups [d.5.2., d.5.3., …) | | |
| Notes: | | |

(duplicate the present table for each outcome)

Sources used to construct the coding form:

Gaffney, H., Jolliffe, D., Eggins, E., Ferreira, J. G., Skinner, G., Ariel, B., & Strang, H. (2024). Protocol: The effect of restorative justice interventions for young people on offending and reoffending: A systematic review. Campbell systematic reviews, 20(2), e1403. https://doi.org/10.1002/cl2.1403

Kethineni, S., Frazier-Kouassi, S., Shigemoto, Y., Jennings, W., Cardwell, S. M., Piquero, A. R., Gay, K., & Sundaravadivelu, D. (2021). PROTOCOL: Effectiveness of parent-engagement programs to reduce truancy and juvenile delinquency: A systematic review. Campbell systematic reviews, 17(3), e1189. https://doi.org/10.1002/cl2.1189
